# Supplementary material for: Airborne observations reveal the fate of the methane from the Nord Stream pipelines
Source: Nat Commun. 2025 Jan 15;16:351. doi: 10.1038/s41467-024-53780-7 (PMC11735838; doi:10.1038/s41467-024-53780-7)
Supplement: Supplementary file 1 — Supplementary Information [file 41467_2024_53780_MOESM1_ESM.pdf]

Supplementary information for 'Airborne observations reveal the fate of the methane from the Nord Stream pipelines'

Friedemann Reum[1], Julia Marshall[1], Henry C. Bittig[2], Lutz Bretschneider[3], Göran Broström[4], Anusha L. Dissanayake[5], Theo Glauch[1, 6], Klaus-Dirk Gottschaldt[1], Jonas Gros[7], Heidi Huntrieser[1], Astrid Lampert[3], Michael Lichtenstern[1], Scot M. Miller[8], Martin Mohrmann[9,4], Falk Pätzold[3], Magdalena Pühl[1], Gregor Rehder[2], and Anke Roiger[1]

[1]Deutsches Zentrum für Luft- und Raumfahrt e.V., Institut für Physik der Atmosphäre, Oberpfaffenhofen, Germany
[2]Leibniz Institute for Baltic Sea Research Warnemünde, Rostock, Germany
[3]Technische Universität Braunschweig, Institute of Flight Guidance, Braunschweig, Germany
[4]University of Gothenburg, Department of Marine Sciences, Gothenburg, Sweden
[5]Independent Researcher, EnvSoln, Badulla, Uva Province, Sri Lanka
[6]University of Heidelberg, Institute of Environmental Physics, Heidelberg, Germany
[7]Independent Researcher, Villars-sur-Glâne, Switzerland
[8]Johns Hopkins University, Baltimore, MD, USA
[9]Voice of the Ocean, Västra Frölunda, Sweden

1 Supplementary methods

Supp. Meth. 1 HELiPOD instrumentation

In addition to the greenhouse gas measurements described in the main text, the sensors described in the following table were onboard the HELiPOD during our campaign to measure meteorological variables, and position and attitude.

Supp. Table 1: Meteorology, position and attitude sensor package onboard the HELiPOD during our campaign. Same as in ref. [1].

Parameter	Sensor
Static pressure	Setra 270
Dynamic pressure	Setra 239
Angle of attack	Setra 239
Angle of sideslip	Setra 239
Static pressure	AMSYS 5812-0150-B
Dynamic pressure	AMSYS 5812-0001-D-B
Angle of attack	AMSYS 5812-0001-D-B
Angle of sideslip	AMSYS 5812-0001-D-B
Temperature	Rosemount 102BW
Temperature	IFF Fine wire set 1
Temperature	IFF Fine wire set 2
Temperature	TSYS TSYS01
Temperature	Vaisala HMP110
Temperature	LICOR LI7500A and LI7700
Temperature	Vaisala HMP110
Humidity	Vaisala HMP110
Humidity	IST P14
Humidity	Buck Lyman-Alpha
Humidity	LICOR LI7500A
Humidity	Buck Lyman-Alpha
Humidity	Vaisala HMP110
Humidity	Meteolabor TP3-ST
Humidity	Meteolabor Snow White
Humidity	GE 1011B
Position	Javad Delta-3
Position	ublox ZED-F9P
Position	iMAR iNAT-M200/SLN
Attitude	iMAR iNAT-M200/SLN
Attitude	Analog Devices ADIS16488
Magnetic field	Magson EnExMag
Radar height	Thomson-CSF ERT 180

Supp. Meth. 2 Inverse model

Supp. Meth. 2.1 Inversion method

The solution that minimises the Bayesian cost function $J(\mathbf{f})$ (Eq. (4) in the main text) is a Gaussian distribution [2] with mean

$$\mathbf{f_{post}} = \left(\mathbf{H}^T\mathbf{R}^{-1}\mathbf{H} + \mathbf{Q}\right)^{-1}\mathbf{H}^T\mathbf{R}^{-1}\boldsymbol{\Delta}\mathbf{c} \tag{S1}$$

and covariance

$$\mathbf{Q_{post}} = \left(\mathbf{Q}^{-1} + \mathbf{H}^T\mathbf{R}^{-1}\right)^{-1}\mathbf{H}, \tag{S2}$$

with transport matrix $\mathbf{H}$, prior flux covariance matrix $\mathbf{Q}$, model-data mismatch matrix $\mathbf{R}$ and mole fraction enhancements $\mathbf{\Delta c}$. The Lagrange multiplier method that restricts the solution to non-negative values modifies both $\mathbf{f_{post}}$ and $\mathbf{Q_{post}}$. The modified posterior covariance matrix $\mathbf{Q_{post}}$ cannot be computed directly [3]. We approximate it by computing the unmodified $\mathbf{Q_{post}}$, i.e. as in Eq. (S2), using the method of ref. [4]. We report the uncertainty of the emissions aggregated over the area where the uncertainty reduction is greater than 0.

The cost function minimization is carried out using the limited-memory Broyden-Fletcher-Goldfarb-Shanno approach (L-BFGS-B[5]).

Supp. Meth. 2.2 Covariance matrices and estimation

The prior flux covariance matrix $\mathbf{Q}$ with spherical covariance model is

$$\mathbf{Q}_{ij} = q^2 \cdot \begin{cases} 1 - 1.5\left(\frac{\text{dist}_{ij}}{d}\right) + 0.5\left(\frac{\text{dist}_{ij}}{d}\right)^3, & \text{dist}_{ij} \leqslant d \\ 0, & \text{dist}_{ij} > d, \end{cases} \tag{S3}$$

where q^2 is the prior flux variance, d is the correlation length and $dist_{ij}$ is the distance between model grid cells i and j.

The maximum likelihood method for covariance parameter estimation is described in ref. [6]. Since we set the prior flux to 0, we minimise the simplified cost function

$$L_{r,q,d} = \frac{1}{2}\ln|\mathbf{HQH}^T + \mathbf{R}| + \frac{1}{2}\mathbf{\Delta c}^T\left(\mathbf{HQH}^T + \mathbf{R}\right)^{-1}\mathbf{\Delta c}, \tag{S4}$$

with the covariance parameters r (model-data mismatch), q (prior uncertainty) and d (correlation length).

We estimate r and q based on the maximum likelihood method for four inversion setups, which use combinations of the two sets of meteorological fields (Meteo A and Meteo B; Supp. Note 2) and the two estimates for the methane background (BG CAMS and BG CO2; Supp. Note 1). While we could also estimate the spatial correlation length d, we find that the estimated values may be too short. The estimated values for d are around 10 km, and in some cases, using this value in inversions produces isolated emission hot spots that may in reality be connected and form a spatially broader emission pattern. Therefore, we use a fixed correlation length of d=15 km. This value is a compromise that simultaneously avoids disconnected emission hot spots while minimising the placement of emissions outside of the area of influence purely by correlation. The estimated covariance parameters are shown in 2.

Supp. Table 2: Estimated covariance parameters used for inversions.

Meteorological fields	Methane background	r [ppb]	q $\left[\frac{\mu\text{mol}}{\text{m}^2\text{s}}\right]$	d [km]
Meteo A	BG CAMS	16.3	0.40	15
Meteo A	BG CO2	16.4	0.36	15
Meteo B	BG CAMS	17.9	0.36	15
Meteo B	BG CO2	17.4	0.29	15

Supp. Meth. 3 Ocean model descriptions

Supp. Meth. 3.1 Ocean model (this study)

near-field model: For simulating how much methane dissolved in the water at the leak sites, we employ the results of the near-field model by Dissanayake et al. [7] ("D23"). The near-field model is comprised of

Supp. Table 3: Short descriptions of the ocean models (same as Table 2 in the main text with an additional column for model M25).

Model	Ocean model (this study)	M25
Dissolved methane at leak locations	Results of near-field model from ref. [7]	Derived from ocean glider data [8]
Total CH_4 dissolved [kt]	10.8	10.8 (9.5-14.7)
Initial vertical distribution of methane dissolved at leak sites	Uniform in mixed layer	Uniform from individual leak depth to surface
Ocean currents	Baltic Sea Physics Analysis and Forecast [9], resolution: 1' latitude, 1'40" longitude, 55 vertical levels	Baltic Sea Physics Analysis and Forecast [9], resolution: 1' latitude, 1'40" longitude, 55 vertical levels
Transport of dissolved methane	Lagrangian, horizontal advection and diffusion in mixed layer. Diffusion calibrated using observations of dissolved methane from DE-SOOP Finnmaid [10] (Supp. Meth. 3.1)	Lagrangian, 3D advection and diffusion
Outgassing	Wanninkhof [11], driven by ERA5 winds	COARE model [12, 13], driven by ERA5 winds
Reference	This study	Ref. [8]

the following components: a model of gas release from the pipes, a bubble plume model, a fountain model and a surface spread model. We briefly summarize the components here. For gas release from the pipes, D23 assumed that 215 kt CH_4 were released from the pipes, based on early estimates [14, 15]. D23 further assumed that the short pipe sections completely empty within 12 hours and the long pipe sections within 6 days. They estimated the curves by fitting two second-order polynomials for each leak for the short and long pipe sections based on the assumed mass of gas released and the duration of release. The use of a second-order polynomial reflects the pressure drop in the pipeline during gas release, similar to the results reported in `https://www.nilu.com/2022/10/improved-estimates-of-nord-stream-leaks/`. D23 then used the Texas A&M Oil Spill Calculator (TAMOC) [16–19] to simulate the multiphase (seawater and gas bubbles) dynamic buoyant plumes formed at the leak locations. The model accounts for ambient water currents, water column stratification, temperature, salinity, and pressure conditions, and the composition of the natural gas. TAMOC simulates mass and heat transfer between the plume water and the gas bubbles for the natural gas constituents and for the oxygen, nitrogen, and argon present in entrained ambient seawater and calculates gas densities and fugacities with the Peng-Robinson equation of state [20, 21]. A fountain [22] and a surface spread model [23, 24] were used to predict the behaviour immediately upon reaching the sea surface. Recent estimates indicate that the total methane content released from the ruptured pipes as well as the flow rates in the first hours may have been higher [25] than earlier estimates, which were used in D23. However, the total amount of dissolved CH_4 derived in D23 from these earlier estimates is close to that derived for model M25 (Supp. Meth. 3.2) from observations of dissolved methane (3).

Transport with ocean currents: The transport of the dissolved methane is modelled using a simple 2D Lagrangian implementation, only taking horizontal transport into account. The mass of methane released per hour, the location of each of the six release points (two per leak), and the radius of the release into the mixed layer (maximum 315 m) are taken from the near-field model from D23 described above. The mass is divided over 101 particles per hour that are evenly spaced within a circle described by the location and radius. The velocity of the particles, u, is decomposed into a mean and turbulent component, $\bar{u}$ and u',

respectively. The mean component (horizontal advection) is implemented using a fourth-order Runge-Kutta scheme with an hourly time step, using the currents from the NEMO Baltic Sea physical model provided by the Copernicus Marine Service [9]. The turbulent diffusion is implemented as a Markov chain, an approach first proposed by refs. [26, 27], and the basis for the STILT Lagrangian atmospheric transport model [28].

The turbulent component of the velocity u' is represented with the following equation:

$$u'(t + \Delta t) = R(\Delta t)u'(t) + u''(t), \tag{S5}$$

where u'' is a random vector and R is an autocorrelation function with the following exponential form:

$$R(\Delta t) = e^{\frac{-\Delta t}{T_{L_i}}} \tag{S6}$$

in which T_{L_i} is the Lagrangian timescale in the horizontal direction. This is a decorrelation timescale that determines the degree to which a particle's movement behaves like a random walk (for $T_{L_i} = 0$) or like determinant advection (for large values of T_{L_i}). The random velocity u'' is defined as:

$$u'' = \lambda[1 - R^2(\Delta t)]^{\frac{1}{2}}, \tag{S7}$$

where λ is a vector of random numbers taken from a Gaussian distribution with mean 0 and standard deviation σ_i, which characterises the spread in the random velocity.

We estimate σ_i using observations of dissolved methane, obtained regularly aboard the DE-SOOP Finnmaid which covered the area 2 times every 3 days. The instrumentation is part of the European Integrated Carbon Observation System Infrastructure (ICOS RI). Methods are described in refs. [29, 30]. For the data in the Bornholm Basin during the time after the Nord stream explosions, the steep CH_4 gradients required application of a time lapse correction. The exact data handling is described in [8], and the data set is available at `https://doi.org/10.18160/K3BM-8YNG`. By comparing the distribution of the modelled to the observed methane plume over time (Supp. Fig. 1), we find $\sigma_i = 0.05$ to be an appropriate value.

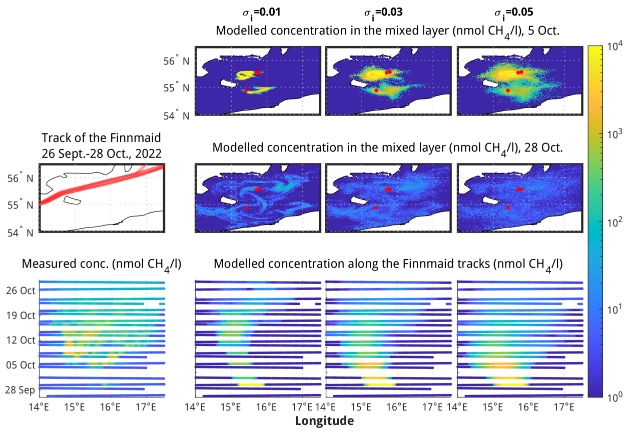

Supp. Fig. 1: Observations of CH_4 dissolved in surface water from the DE-SOOP Finnmaid (lower left panel) as measured along its track (middle left panel) from 26 Sept.-28 Oct., 2022. The next three columns show modelled methane concentrations in the mixed layer in our ocean model using different values for the dispersion factor σ_i: 0.01, 0.03, and 0.05. The top row shows the concentrations on the day of the flights (5 Oct.), the middle row shows concentrations on the last day of the ship measurements considered here (28 Oct.), and the bottom row shows the modelled concentrations along the track of the SOOP for the full period, smoothed with a one-hour moving average. The locations of the leaks are marked in red. As σ_i increases, the shape of the distribution of the modelled CH_4 concentrations approaches the spatial extent of the methane enhancements in the measurements.

Several studies have estimated Lagrangian time scales in the surface ocean based upon drifter and float data [31–33], and a typical value for T_{L_i} is found to be approximately two days for the open ocean. An analysis

in the Baltic found considerably shorter Langrangian time scales, ranging from half a day to approximately two days, with the peak of the mean probability density function between 21 and 28 hours [34]. For simplicity, a value of 24 hours has been chosen for the following simulations. To simulate the Markov chain well, it is necessary to adequately resolve the autocorrelation of u' from the previous time step, i.e. the previous link in the Markov chain. This can be ensured by choosing a time step Δt that is smaller than one tenth the Lagrangian timescale T_{L_i} [35]. Thus, a time step of one hour is sufficiently short.
Outgassing to the air: Outgassing in our ocean model is determined by a piston velocity model, which is driven by the concentration difference of CH_4 in the surface water (C_{water}) and the concentration that would be in equilibrium with the atmosphere (C_{eq}):

$$F = k \cdot (C_{\text{water}} - C_{\text{eq}}) \tag{S8}$$

where the flux F ($\text{mol}\,\text{m}^{-2}\,\text{s}^{-1}$) is calculated as a product of the gas transfer velocity k ($\text{m}\,\text{s}^{-1}$) and this concentration difference. To get the concentration from the mass attached to each Lagrangian particle, we assume that the mass is evenly distributed within the mixed layer, the depth of which is taken from ref. [9]. The horizontal grid spacing is $\left({}^{1}/_{60}\right)^{\circ}$ in the latitudinal direction and $\left({}^{1}/_{36}\right)^{\circ}$ in the longitudinal direction. The equilibrium concentration C_{eq} is approximately $3.2\,\text{nmol CH}_4\,\text{l}^{-1}$, and can be neglected given the large supersaturations involved [7].

The gas transfer velocity k is calculated according to the equations given in ref. [11]):

$$k \left[\frac{\text{cm}}{\text{h}}\right] = 0.251 \cdot u^2 \cdot \left(\frac{\text{Sc}}{660}\right)^{-0.5} \tag{S9}$$

Here u is the 10-m wind speed in meters per second, taken from ERA5 reanalyses [36]. The Schmidt Number (Sc) is calculated using the parameterisation given in ref. [11], based on the water temperature from the ocean model for both fresh water and saltwater (salinity 35 ‰), and then an average value is taken, linearly weighted according to the salinity of the mixing layer in the model.

Supp. Meth. 3.2 Ocean model (M25)

Mohrmann et al. [8] conducted a study, continuously monitoring CH_4 concentrations for three months after the leaks. They employed both SeaExplorer ocean gliders and the DE-SOOP Finnmaid data described in Supp. Meth. 3.1. The SeaExplorers were strategically positioned near the leak sites, enabling them to capture the variations in dissolved methane across different depths and horizontal distribution within a relatively short transect of approximately 20 kilometers in the vicinity of the northern leak sites. The DE-SOOP Finnmaid collected surface measurements on a broader spatial scale, thus adding estimates of the horizontal spread of the methane plume. These observations, obtained through ferry and glider platforms, were instrumental in characterising the plume's structure and establishing the initial conditions for their model. The model, which is based on the OpenDrift module ChemicalDrift [37] simulates advection, diffusion and outgassing. Microbial oxidation is not included in M25, and a sensitivity test shows that its effect on emissions on 5 October 2022 was negligible [8]. The input data used for the 3D-advection and mixed layer depth estimates as well as the wind (ERA5) are identical to the ones used in our ocean model (Supp. Meth. 3.1). A detailed model description can be found in ref. [8]. The spatially integrated results (Supp. Fig. 12) are obtained with the model runs shown in ref. [8]. The spatially resolved results (Supp. Fig. 10 and 5) are obtained with a model run that is similar to the central estimate of ref. [8] with more Lagrangian particles, which improves the robustness of spatially resolved results. The minor deviations in dissolution rate and dissolved mass compared to the run used for the spatially integrated results. The reason for choosing this run for analysing the spatial distribution of the emissions is that.

2 Supplementary notes

Supp. Note 1 Atmospheric methane background mole fractions

Here we show the results for the two estimation methods for atmospheric methane background introduced in the Methods in the main text, "BG CAMS" and "BG CO2".

Observations. The lowest methane mole fractions observed within the boundary layer were 2051.8±1 ppb, measured over a period of 44 seconds during Flight 2 (15:08:06-15:08:50 UTC) across a distance of 1.6 km. This took place close to the northwestern edge of the flight path (55.33°N, 15.16°E), i.e. at the location furthest upwind of the leaks. Similarly low methane mole fractions were observed at portions of the western transfer leg of Flight 2 (2054 ppb, 15:24-15:25 UTC), and at the southern end of the eastern transfer leg (2057 ppb, 13:46-13:51 UTC). For the locations, see also Supp. Fig. 3.

BG CAMS. We obtain the BG CAMS set of methane background mole fractions by sampling the CAMS dataset [38] along the flight path and fitting an offset to match the lowest observed mole fractions (2051.8 ppb, observed in the northwest). The offset is -7 ppb. Thus, "BG CAMS" matches the observations in the northwest by design. It is also close to the observations at the southern end of the eastern transfer leg (Supp. Fig. 2). In between and during Flight 1, all methane observations are higher, yielding spatially widespread enhancements over the background. Therefore, BG CAMS represents a lower bound for the methane background. Mean and standard deviation of BG CAMS are 2059.4±2.3 ppb for Flight 1 and 2054.2±2.5 ppb for Flight 2.

BG CO2. For obtaining BG CO2, we flag a set of methane observations in the troughs between peaks as possible background values, as an upper limit for the methane background (indicated in Supp. Fig. 2-Supp. Fig. 4). These are used as supporting points to obtain BG CO2 as defined in Eq. (2) in the main text (Supp. Fig. 2 and Supp. Fig. 3). BG CO2 closely follows an arc spanned by the supporting points during Flight 2, while overestimating the background near the profile close to the southern leak during Flight 1 (Supp. Fig. 2, 9:00-9:05 UTC). The reason is likely an unknown upwind CO_2 source, causing higher observed CO_2 but no methane enhancement. Therefore, we remove that profile in inversions that use BG CO2 (17 of the 481 data points). Mean and standard deviation of BG CO2 are 2102±5 ppb during Flight 1 and 2065±9 ppb during Flight 2.

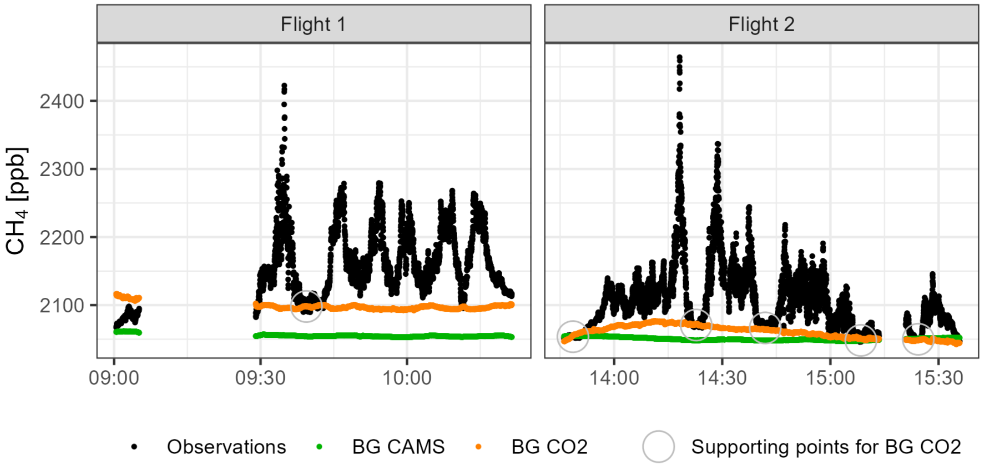

Supp. Fig. 2: Time series of methane observations used to estimate emissions and the two estimates of background mole fractions. Plots of the datasets used to derive the background estimates (CAMS and observations) are shown in Supp. Fig. 4.

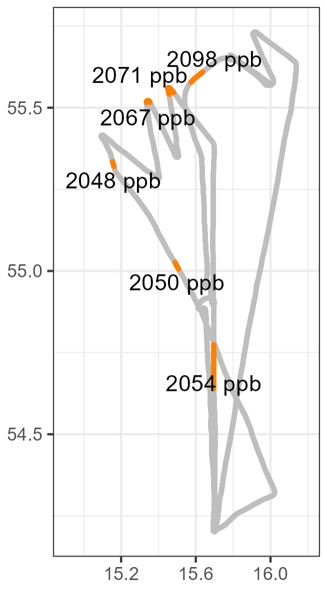

Supp. Fig. 3: Locations of supporting points used in the fit of BG CO2.

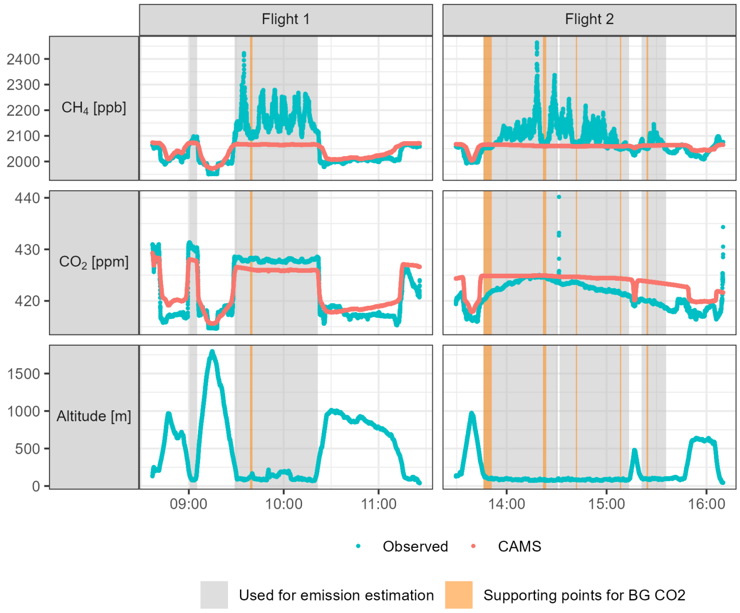

Supp. Fig. 4: CO_2 and CH_4, measured and sampled from CAMS [38].

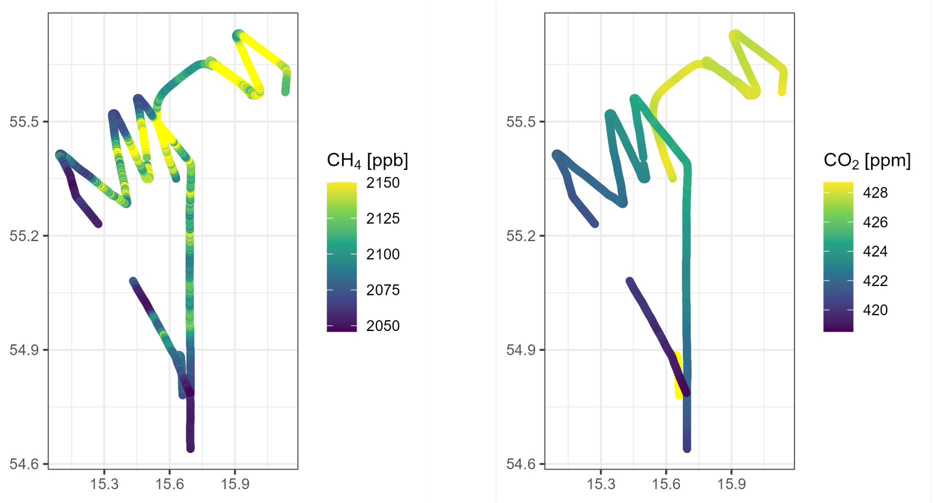

Supp. Fig. 5: Measured CH_4 and CO_2. In order to better visualize the correlation between the CH_4 peaks (see also the "BG CO2" curve in Supp. Fig. 2), the colour scale for CH_4 cuts off the peaks and the colour scale for CO_2 cuts off the high values observed during the profile at the southern leak during Flight 1.

Supp. Note 2 Evaluation of modelled meteorology

The estimation of the emission footprints requires accurately modelled meteorological fields. Important parameters are the wind fields as well as the boundary layer height, as they have a direct impact on the atmospheric methane distribution and the STILT footprints. In the following we compare the modelled meteorology to the meteorological observations along the flight path, specifically winds and temperature profiles.

Supp. Note 2.1 Wind speed and wind direction

Supp. Fig. 6 and Supp. Fig. 7 show the wind direction and wind speed for the measurements and the modelled meteorological fields, Meteo A and Meteo B. The visible jumps in wind speed and direction are mostly related to changes in the flight altitude, as shown in the bottom panels. The further discussion focuses on the flight portions inside the boundary layer, as these are the data used for emission estimation.

Wind speed and direction in the boundary layer were rather constant. Overall, the modelled wind fields are mostly consistent within the measured variabilities. The deviations are summarised in the following. For the wind direction, we observe small differences between the measurements and Meteo A and Meteo B. In Flight 1, the deviation in Meteo A is a constant shift of -6° (in the time interval t_0: 2022-10-05 09:29:00 UTC to t_1: 2022-10-05 10:22:00 UTC). In Flight 2, the deviation roughly follows a linear function of the form 9°/h·(t - t_0) - 8° (over the time interval t_0: 2022-10-05 14:13:00 UTC to t_1: 2022-10-05 15:14:05 UTC). In Meteo B, the shift in Flight 1 is reduced to -3°, which is the result of assimilating the observations in WRF. During Flight 2 Meteo B does, however, not show any improvement in the wind direction compared to Meteo A.

Supp. Note 2.2 Height of the planetary boundary layer

To compare the planetary boundary layer (PBL) height from the WRF model and the measurements, we consider the vertical profiles of the virtual potential temperature, which shows a characteristic increase at the capping inversion height (e.g. ref. [39]). Along the flight path, 12 descents and ascents suitable for determining vertical temperature profiles are available.

The profiles are shown in Supp. Fig. 8. Generally, the PBL was neutral to unstably stratified due to the relatively warm water surface temperature of 15.1°C and the cooler air masses with an air temperature of ∼14.6°C advected from the southwest. As expected, we observe a clear diurnal cycle. There is a capping inversion in the morning at just below 300 m. During the morning, the height of the PBL increases and the capping inversion becomes weaker.

There is little difference in the profiles between Meteo A and Meteo B. Both model runs yield a low bias in the virtual potential temperature and less pronounced capping inversions than observed. Systematically lower virtual potential temperature in WRF compared to observations have been observed during airborne measurements above the North Sea as well [40, 41]. In general, however, the profiles and their evolution throughout the day are compatible to the observations, indicating that both WRF runs capture the evolution of the turbulent structure of the atmosphere. Based on the WRF profiles, PBL heights are derived in STILT for footprint calculation, restricted to WRF model layers (horizontal lines). In most cases, the modelled PBL heights are close to observed pronounced capping inversions, but the comparison is more difficult in the afternoon due to the weakening of the capping inversions. In all cases, modelled or observed PBL heights are below about 600 m. The modelled PBL height drops to below 400 m in the profiles at 15:19 UTC and 15:49 UTC, which is in the southwest of our flight pattern. These values may be too low considering that we do not expect a collapse of boundary layer turbulence that early in the day. Such an underestimation of the PBL height may lead to an underestimation of emissions upwind of this area. However, this potential local bias in the emissions has a limited impact since observed methane enhancements during this period were low compared to other places in our domain; hence emissions estimated from these data contribute little to the total.

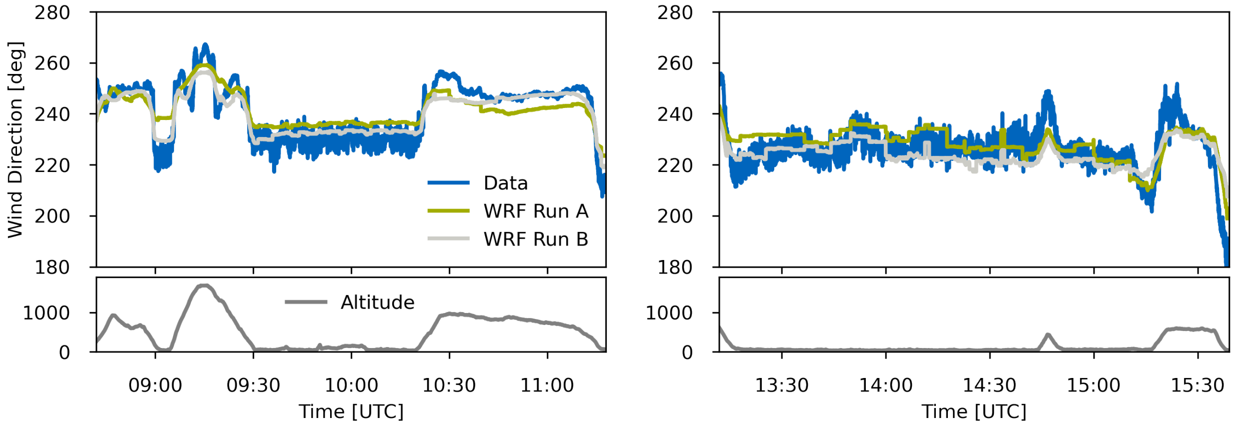

Supp. Fig. 6: Comparison between the measured wind direction and Meteo A/B for Flight 1 (left) and Flight 2 (right). The bottom panels show the flight altitude.

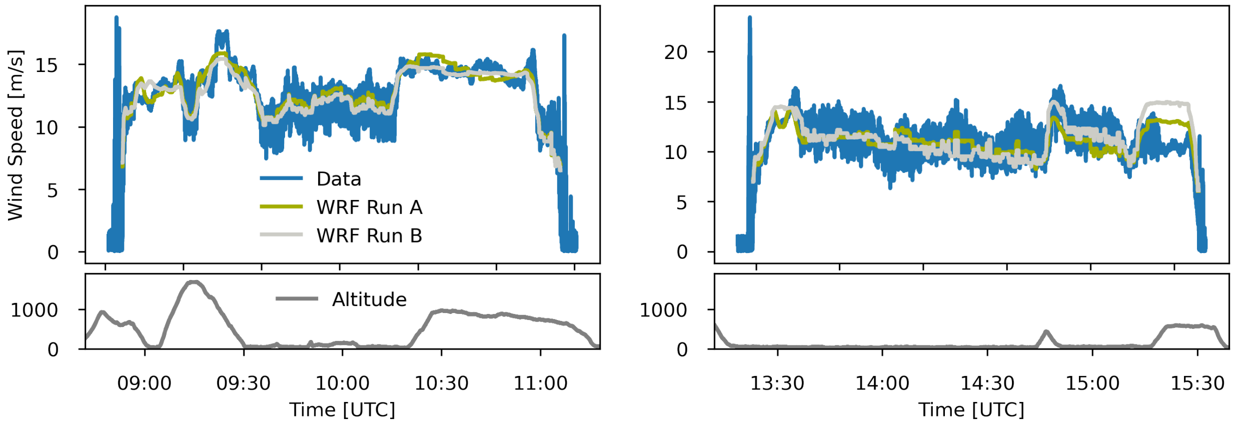

Supp. Fig. 7: Comparison between the measured wind speed and the WRF model for Flight 1 (left) and Flight 2 (right). The bottom panels show the flight altitude.

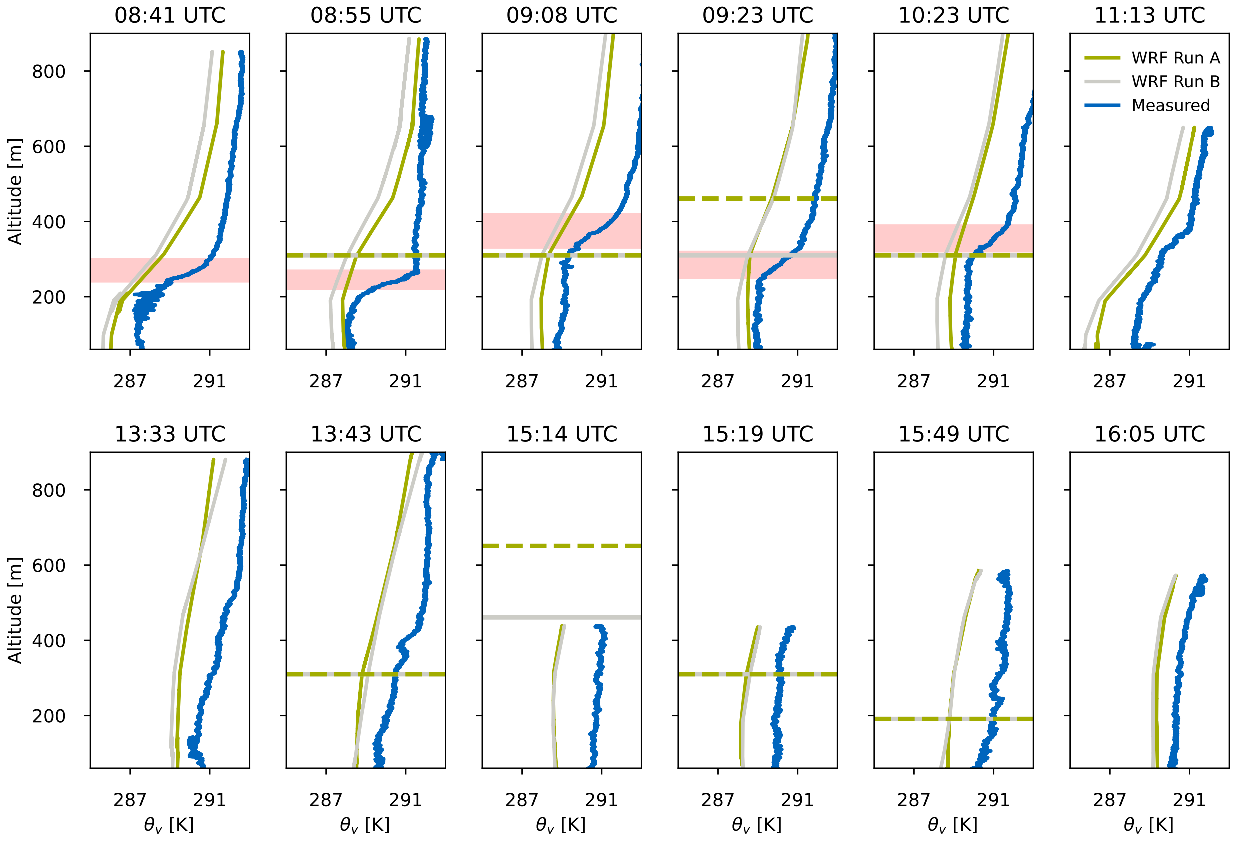

Supp. Fig. 8: Vertical profiles of the virtual potential temperature from WRF and the in situ measurements. The red shaded bands visualize the observed capping inversion in the cases where it is pronounced. The horizontal lines represent the PBL height calculated by STILT and used in the footprint estimation. Cases without horizontal lines are outside of the area of interest, so we did not run the STILT model for them.

Supp. Note 3 Breakdown of uncertainty in emissions

We perform inversions based on the two estimates of background methane mole fractions (see Methods in the main text) and two sets of meteorological fields (Supp. Note 2), for a total of four inversions. In addition, we compute scaling factors for the posterior emissions from the inversions such that methane mole fractions modelled with WRF-GHG (see Methods in the main text) instead of STILT fit the atmospheric data, which illuminates discrepancies between the Eulerian and the Lagrangian transport approach (see Methods in the main text). This brings the number of emission estimates to eight. The total methane emissions are summarised in 4. The inversion results are 23-48 $t\,h^{-1}$. With these emissions, WRF-GHG slightly overestimates the observations, and using the simple scaling factor approach, emissions of 19-47 $t\,h^{-1}$ are compatible with the observations. Therefore, the total range of emission estimates is 19-48 $t\,h^{-1}$.

Meteo B yields emissions that are on average ~30 % lower than those from Meteo A. The difference is mostly related to modelled PBL height, which is on average ~ 20 % lower along the flight track in Meteo B than in Meteo A. However, in most cases, the PBL heights from both meteorological fields agree with capping inversions observed during vertical profiles in the flight tracks (Supp. Fig. 8).

Supp. Table 4: Total methane emissions in [$t\,h^{-1}$]. The uncertainties of the inversions are the Bayesian posterior uncertainty. The WRF-GHG results are obtained by applying a scaling factor to the WRF-STILT inversion results as described in the Methods in the main text.

Meteorological fields	Methane background	WRF-STILT inversion	WRF-GHG scaled
Meteo A	BG CAMS	48±8	47±8
Meteo A	BG CO2	34±7	32±7
Meteo B	BG CAMS	34±8	29±7
Meteo B	BG CO2	23±6	19±5

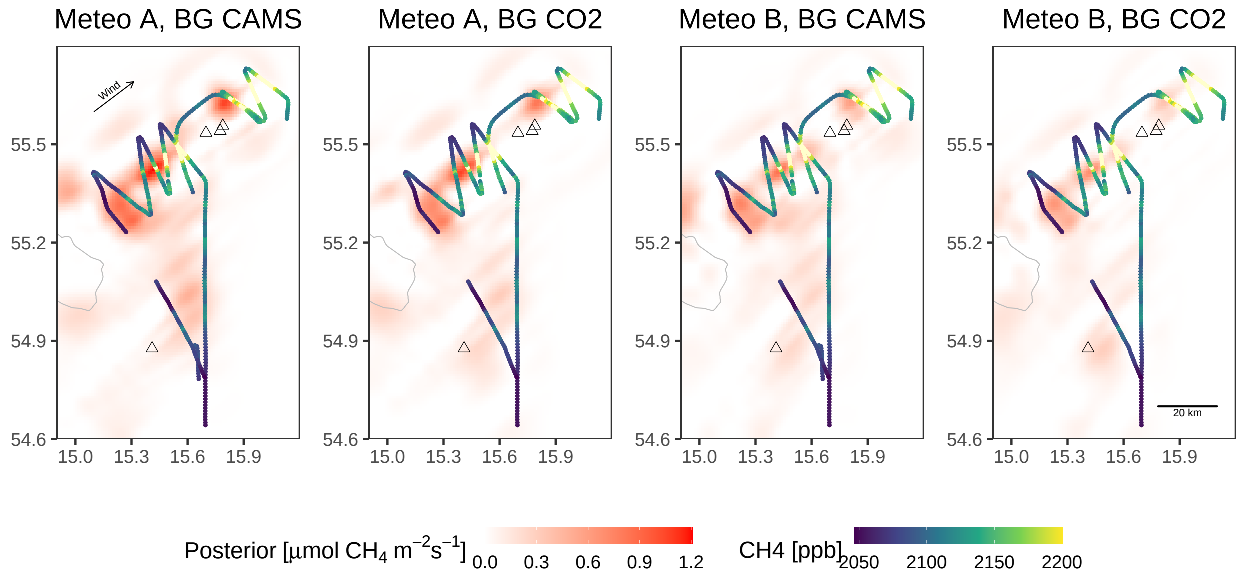

Supp. Fig. 9: Maps of methane measurements and emissions retrieved in the four inversions. Fig. 3a in the main text is the same as the second from the left here, labelled "Meteo A, BG CO2".

Supp. Note 4 Results of ocean models

Here, we provide results from the ocean model M25 in context with results from our model shown in the main text.

Supp. Table 5: Summary of methane emissions on 5 October 2022 from ocean models in comparison with inversion results and the atmospheric data used in the inversions (same as Table 1 in the main text with an additional column for model M25).

Metric	Inversion	Ocean model (this study)	M25
Emissions			
Total emissions [t h^{-1}]	19-48	32	13 (11-18)
Correlation with emissions from inversion	0.79-1	0.25-0.34	-0.01-0.03
Area with 90% of total emissions [10^9 m^2]	3.2-3.7	4.7	2.6
Modelled atmospheric methane mole fractions			
Correlation with atmospheric data	0.92-0.95	0.41-0.64	0.06-0.20
Mean bias [ppb]	-0.7-0.4	-33-(-1)	-62-(-41)
Mean enhancement [ppb]	51-70	38-50	9-10

Supp. Table 6: Correlations among the emissions estimated using airborne data and the ocean models. The ranges represent the four inversions.

	Inversion	Ocean model (this study)	M25
Inversion	0.79-1	0.25-0.34	-0.01-0.03
Ocean model (this study)		1	0.32
M25			1

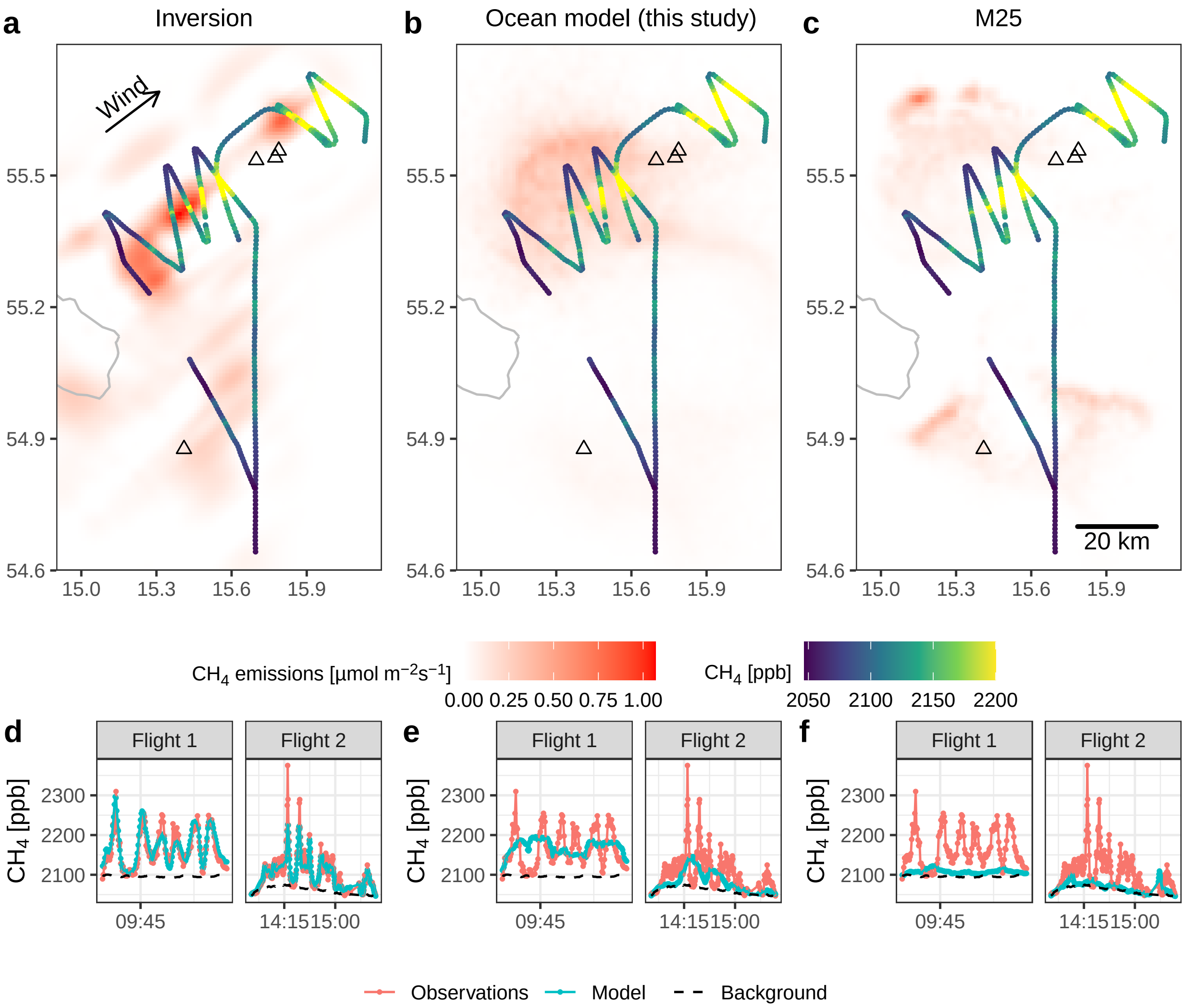

Supp. Fig. 10: Same as Fig. 3 in the main text, but with the addition of panels c and f for model M25.

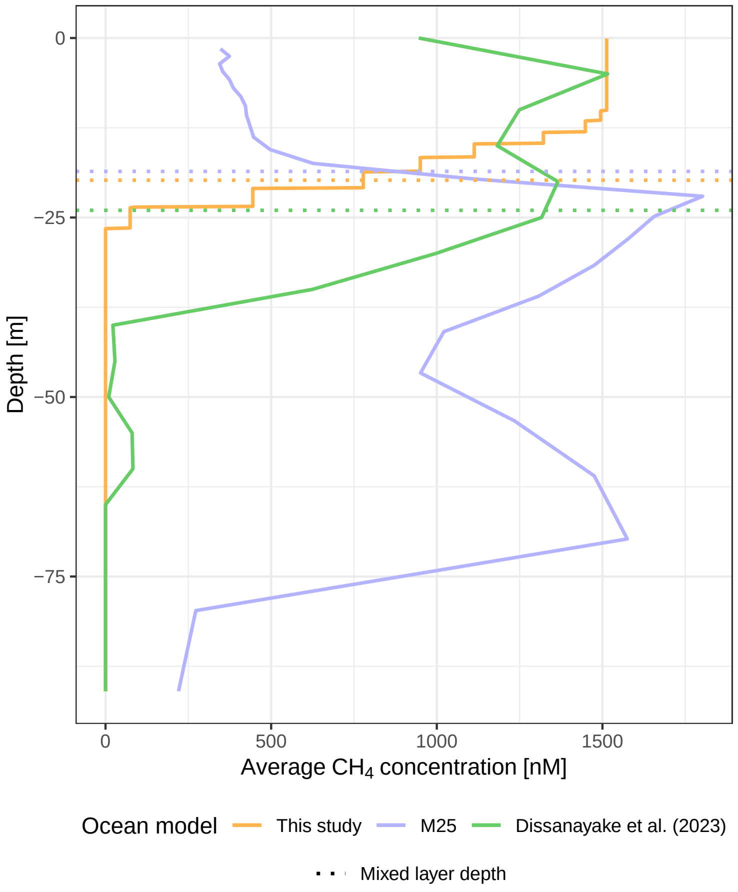

Supp. Fig. 11: Profiles of the dissolved methane concentration in our ocean model, M25 and the ocean model from Dissanayake et al. [7]. The latter model is included to demonstrate that the 2D simplification in our ocean model has little impact on the concentration of dissolved methane in the mixed layer on 5 October 2022. Concentrations from 5 October 2022 12 UTC (i.e. close the middle of the observation period) are averaged over a box that contains most of the emissions from all models (lower left: 15.0°E, 55.2°N, upper right: 16.0°E, 55.8°N).

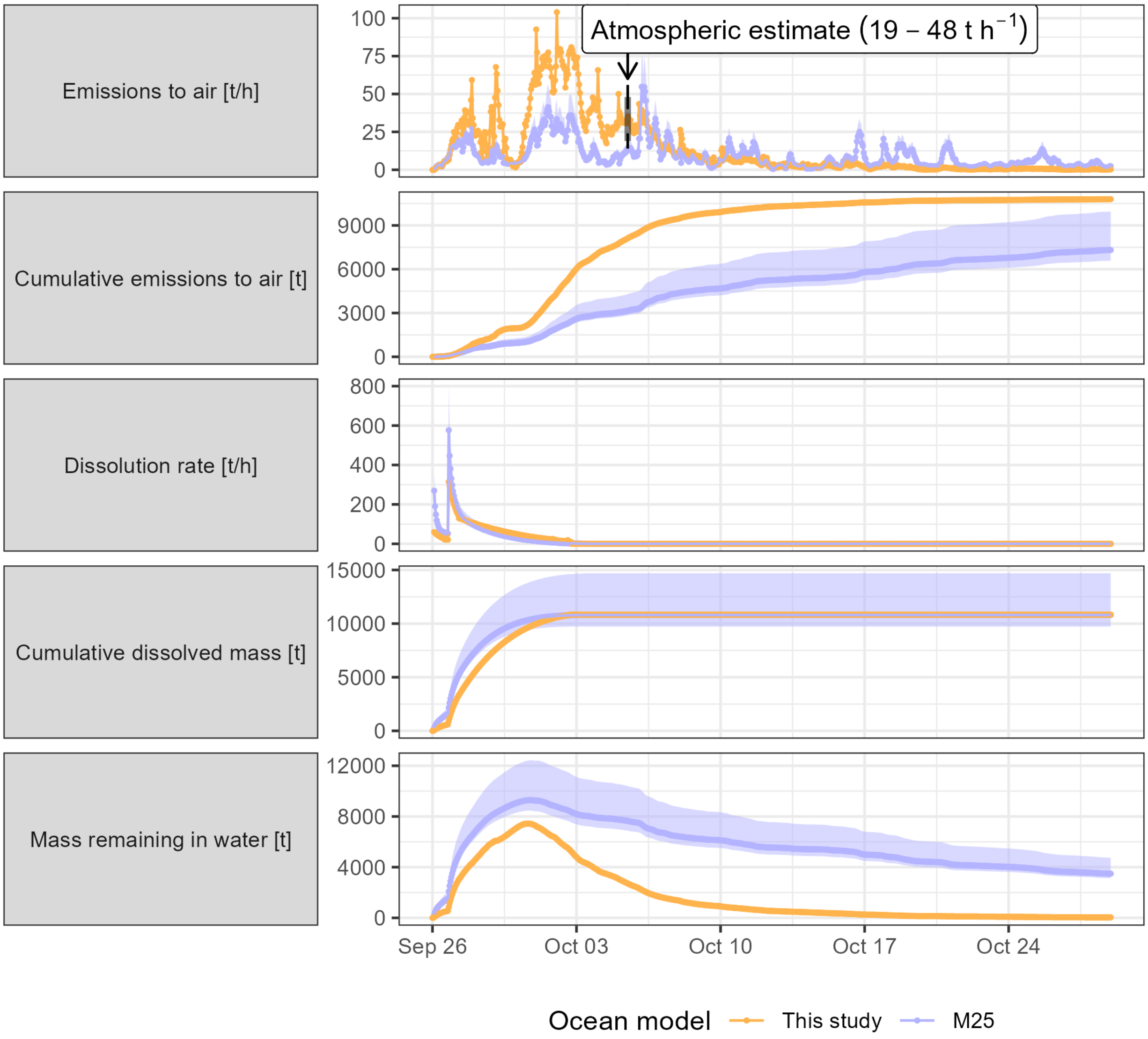

Supp. Fig. 12: Time series of ocean model inputs (dissolved mass) and outputs (emissions to the atmosphere and remaining mass in water). For comparison, the emissions obtained from the atmospheric data are plotted over the period to which they were sensitive. The error bars indicate the 1-σ Bayesian posterior uncertainty of the lower and upper bound, respectively.

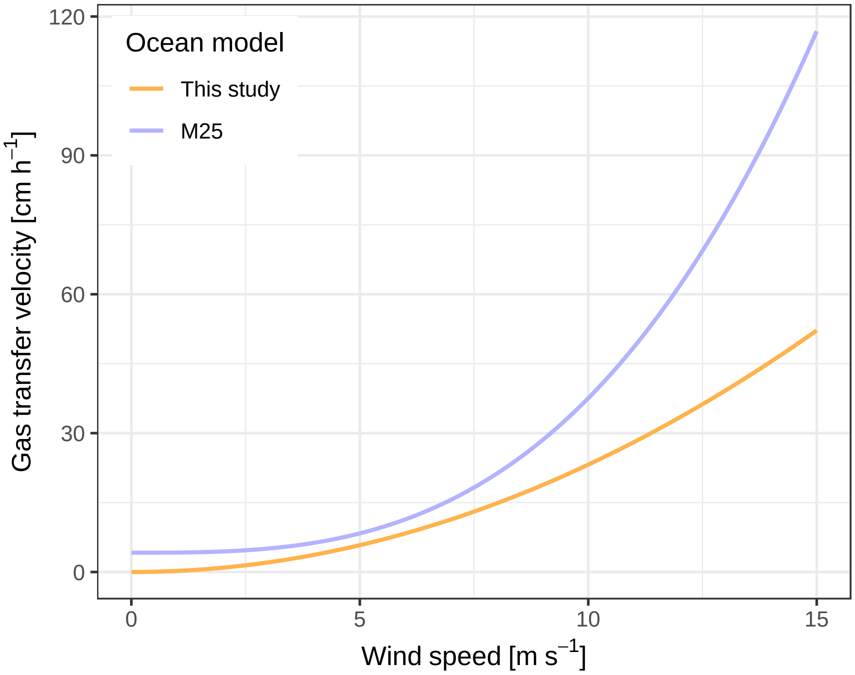

Supp. Fig. 13: Gas transfer velocity parameterisations used in our ocean model [11] and in M25 [13]. Shown here are values for a range of wind speeds at a constant water temperature of 16°C and a salinity of $8\,\mathrm{g\,kg^{-1}}$.

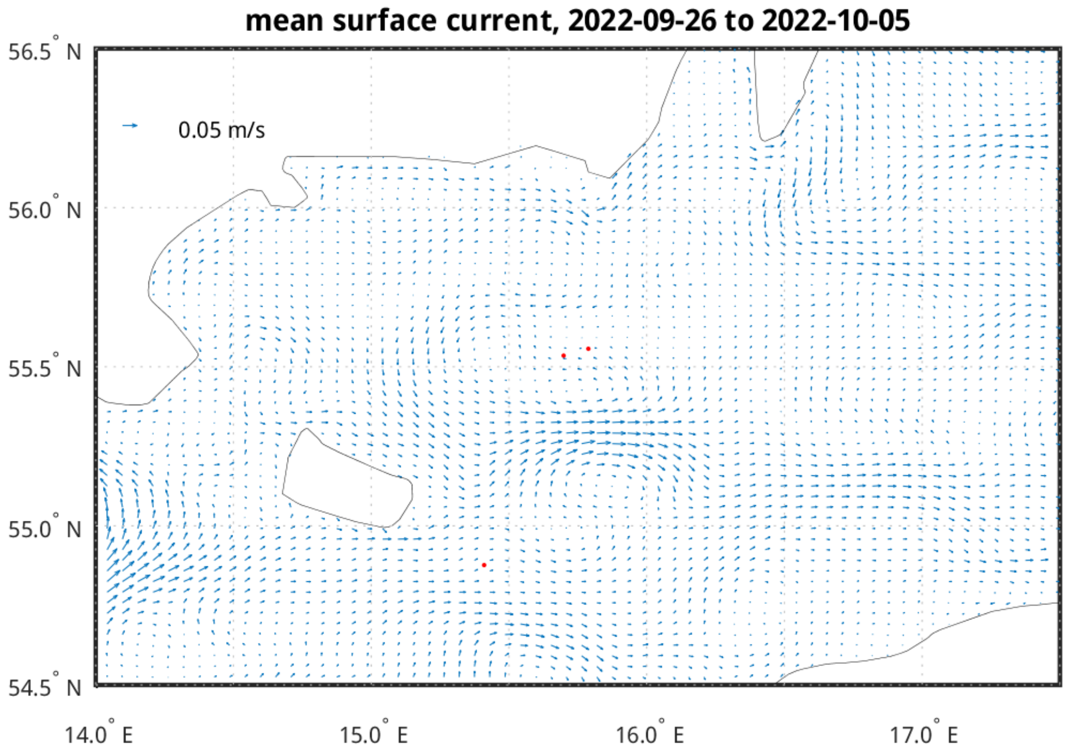

Supp. Fig. 14: Mean modelled surface currents used in our ocean model [9]. Bornholm Basin, 26 September to 5 October 2022. The three largest Nord Stream leaks are shown as read dots. The center of the Bornholm Basin is close to 55°N, 15.5°E in the model.

References

1. Pätzold, F. *et al.* HELiPOD—Revolution and evolution of a helicopter-borne measurement system for multidisciplinary research in demanding environments. *Elem Sci Anth* **11.** ISSN: 2325-1026. https://doi.org/10.1525/elementa.2023.00031 (2023).
2. Tarantola, A. *Inverse Problem Theory and Methods for Model Parameter Estimation* https://doi.org/10.1137/1.9780898717921 (Society for Industrial and Applied Mathematics, Jan. 2005).
3. Miller, S. M., Michalak, A. M. & Levi, P. J. Atmospheric inverse modeling with known physical bounds: an example from trace gas emissions. *Geoscientific Model Development* **7,** 303–315. https://doi.org/10.5194/gmd-7-303-2014 (Feb. 2014).
4. Yadav, V. & Michalak, A. M. Improving computational efficiency in large linear inverse problems: an example from carbon dioxide flux estimation. *Geoscientific Model Development* **6,** 583–590. https://doi.org/10.5194/gmd-6-583-2013 (May 2013).
5. Byrd, R. H., Lu, P., Nocedal, J. & Zhu, C. A Limited Memory Algorithm for Bound Constrained Optimization. *SIAM Journal on Scientific Computing* **16,** 1190–1208. https://doi.org/10.1137/0916069 (Sept. 1995).
6. Michalak, A. M. *et al.* Maximum likelihood estimation of covariance parameters for Bayesian atmospheric trace gas surface flux inversions. *Journal of Geophysical Research* **110.** https://doi.org/10.1029/2005jd005970 (2005).
7. Dissanayake, A. L., Gros, J., Drews, H. J., Nielsen, J. W. & Drews, A. Fate of Methane from the Nord Stream Pipeline Leaks. *Environmental Science & Technology Letters.* https://doi.org/10.1021/acs.estlett.3c00493 (Sept. 2023).
8. Mohrmann, M., Biddle, L. C., Rehder, G., Bittig, H. & Queste, B. Y. Nord Stream methane leaks spread across 14% of Baltic waters. *Nature Communications* (2025).
9. CMEMS. *Baltic Sea Physics Analysis and Forecast. E.U. Copernicus Marine Service Information (CMEMS). Marine Data Store (MDS).* en. 2020. https://doi.org/10.48670/MOI-00010.
10. Bittig, H., Glockzin, M. & Rehder, G. *ICOS-Germany SOOP Finnmaid surface sea water CH_4 observations in the Baltic after the 2022 Nord Stream pipeline leaks* 2023. https://doi.org/10.18160/K3BM-8YNG.
11. Wanninkhof, R. Relationship between wind speed and gas exchange over the ocean revisited. *Limnology and Oceanography: Methods* **12,** 351–362. https://doi.org/10.4319/lom.2014.12.351 (June 2014).
12. Johnson, M. T. A numerical scheme to calculate temperature and salinity dependent air-water transfer velocities for any gas. *Ocean Science* **6,** 913–932. ISSN: 1812-0792. https://doi.org/10.5194/os-6-913-2010 (Oct. 2010).
13. Schwarzenbach, R. P., Gschwend, P. M. & Imboden, D. M. *Environmental organic chemistry, Third Edition* 592–600. ISBN: 978-1-118-76723-5 (John Wiley & Sons, 2017).
14. IMEO. *Estimate of Total Methane Emissions from the Nord Stream Gas Leak Incident - Draft Working Paper* 2023. https://wedocs.unep.org/20.500.11822/41838.
15. Chen, X. & Zhou, T. Negligible Warming Caused by Nord Stream Methane Leaks. *Advances in Atmospheric Sciences* **40,** 549–552. ISSN: 1861-9533. https://doi.org/10.1007/s00376-022-2305-x (Nov. 2022).
16. Socolofsky, S. A. *et al. Texas A&M Oilspill Calculator (TAMOC) modeling suite for subsea spills* in *Proceedings of the thirty-eighth AMOP technical seminar* (2015), 153–168. https://infoscience.epfl.ch/record/209022.
17. Gros, J. *et al.* Petroleum dynamics in the sea and influence of subsea dispersant injection during Deepwater Horizon. *Proceedings of the National Academy of Sciences* **114,** 10065–10070. ISSN: 1091-6490. https://doi.org/10.1073/pnas.1612518114 (Aug. 2017).
18. Dissanayake, A. L., Gros, J. & Socolofsky, S. A. Integral models for bubble, droplet, and multiphase plume dynamics in stratification and crossflow. *Environmental Fluid Mechanics* **18,** 1167–1202. ISSN: 1573-1510. https://doi.org/10.1007/s10652-018-9591-y (May 2018).

19. Jun, I. *A Numerical Model For Hydrocarbon Bubbles from Natural Seeps Within Hydrate Stability Zone, Texas A&M University, College Station, Texas* PhD thesis (Texas A&M University, 2018). https://hdl.handle.net/1969.1/174345.

20. Robinson, D. B. & Peng, D.-Y. *The characterization of the heptanes and heavier fractions for the GPA Peng-Robinson programs* Research Report 28 (Gas processors association, Tulsa, 1978).

21. Peng, D.-Y. & Robinson, D. B. A new two-constant equation of state. *Industrial & Engineering Chemistry Fundamentals* **15,** 59–64. ISSN: 1541-4833. https://doi.org/10.1021/i160057a011 (Feb. 1976).

22. Friedl, M. & Fanneløp, T. Bubble plumes and their interaction with the water surface. *Applied Ocean Research* **22,** 119–128. ISSN: 0141-1187. https://doi.org/10.1016/s0141-1187(99)00022-x (Apr. 2000).

23. Brevik, I. & Kristiansen, Ø. The flow in and around air-bubble plumes. *International Journal of Multiphase Flow* **28,** 617–634. ISSN: 0301-9322. https://doi.org/10.1016/S0301-9322(01)00077-5 (Apr. 2002).

24. Dissanayake, A. L., Yapa, P. D. & Nakata, K. Modelling of hydrothermal vent plumes to assess the mineral particle distribution. *Journal of Hydraulic Research* **52,** 49–66. ISSN: 1814-2079. https://doi.org/10.1080/00221686.2013.854845 (Jan. 2014).

25. Harris, S. J. et al. Methane emissions from the Nord Stream subsea pipeline leaks. *Nature* (2025).

26. Obukhov, A. in *Advances in Geophysics* 113–116 (Elsevier, 1959). https://www.sciencedirect.com/science/article/pii/S0065268708600989.

27. Smith, F. B. Conditioned particle motion in a homogeneous turbulent field. *Atmospheric Environment (1967)* **2,** 491–508. ISSN: 0004-6981. https://www.sciencedirect.com/science/article/pii/0004698168900425 (1968).

28. Lin, J. C. A near-field tool for simulating the upstream influence of atmospheric observations: The Stochastic Time-Inverted Lagrangian Transport (STILT) model. *Journal of Geophysical Research* **108,** ACH 2–1–ACH 2–17. https://doi.org/10.1029/2002jd003161 (2003).

29. Gülzow, W., Rehder, G., Schneider, B., Deimling, J. S. v. & Sadkowiak, B. A new method for continuous measurement of methane and carbon dioxide in surface waters using off-axis integrated cavity output spectroscopy (ICOS): An example from the Baltic Sea. *Limnology and Oceanography: Methods* **9,** 176–184. ISSN: 1541-5856. https://doi.org/10.4319/lom.2011.9.176 (Apr. 2011).

30. Jacobs, E. *et al.* Upwelling-induced trace gas dynamics in the Baltic Sea inferred from 8 years of autonomous measurements on a ship of opportunity. *Biogeosciences* **18,** 2679–2709. ISSN: 1726-4189. https://doi.org/10.5194/bg-18-2679-2021 (Apr. 2021).

31. McClean, J. L., Poulain, P.-M., Pelton, J. W. & Maltrud, M. E. Eulerian and Lagrangian Statistics from Surface Drifters and a High-Resolution POP Simulation in the North Atlantic. *Journal of Physical Oceanography* **32,** 2472–2491. https://journals.ametsoc.org/view/journals/phoc/32/9/1520-0485_2002_032_2472_ealsfs_2.0.co_2.xml (2002).

32. LaCasce, J. H. Statistics from Lagrangian observations. *Progress in Oceanography* **77,** 1–29. ISSN: 0079-6611. https://www.sciencedirect.com/science/article/pii/S0079661108000232 (2008).

33. Garraffo, Z. D., Mariano, A. J., Griffa, A., Veneziani, C. & Chassignet, E. P. Lagrangian data in a high-resolution numerical simulation of the North Atlantic: I. Comparison with in situ drifter data. *Journal of Marine Systems* **29.** Three-Dimensional Ocean Circulation: Lagrangian measurements and diagnostic analyses, 157–176. ISSN: 0924-7963. https://www.sciencedirect.com/science/article/pii/S092479630100015X (2001).

34. Kjellsson, J. & Döös, K. Surface drifters and model trajectories in the Baltic Sea. *Boreal Environment Research* **17,** 447–459. https://hdl.handle.net/10138/229941 (2012).

35. Wilson, J. D. & Zhuang, Y. Restriction on the timestep to be used in stochastic Lagrangian models of turbulent dispersion. *Boundary-Layer Meteorology* **49,** 309–316. https://doi.org/10.1007/bf00120975 (Nov. 1989).

36. Hersbach, H. *et al. Complete ERA5 from 1940: Fifth generation of ECMWF atmospheric reanalyses of the global climate. Copernicus Climate Change Service (C3S) Data Store (CDS).* 2017. https://doi.org/10.24381/cds.143582cf.

37. Aghito, M. *et al.* ChemicalDrift 1.0: an open-source Lagrangian chemical-fate and transport model for organic aquatic pollutants. *Geoscientific Model Development* **16,** 2477–2494. ISSN: 1991-9603. https://gmd.copernicus.org/articles/16/2477/2023/ (May 2023).

38. CAMS. *CAMS global inversion-optimised greenhouse gas fluxes and concentrations. Version: v22r1, optimized using surface-air samples* 2023. https://ads.atmosphere.copernicus.eu/cdsapp#!/dataset/cams-global-greenhouse-gas-inversion.

39. Zhang, Y. *et al.* On the computation of planetary boundary-layer height using the bulk Richardson number method. *Geoscientific Model Development* **7,** 2599–2611. https://doi.org/10.5194/gmd-7-2599-2014 (Nov. 2014).

40. Rausch, T. *et al.* Wind Lidar and Radiosonde Measurements of Low-Level Jets in Coastal Areas of the German Bight. *Atmosphere* **13.** ISSN: 2073-4433. https://www.mdpi.com/2073-4433/13/5/839 (2022).

41. Siedersleben, S. K. *et al.* Evaluation of a Wind Farm Parametrization for Mesoscale Atmospheric Flow Models with Aircraft Measurements. *Meteorologische Zeitschrift* **27,** 401–415. http://doi.org/10.1127/metz/2018/0900 (Dec. 2018).
